# GC-MS and LC-DAD-MS Phytochemical Profiling for Characterization of Three Native *Salvia* Taxa from Eastern Mediterranean with Antiglycation Properties

**DOI:** 10.3390/molecules28010093

**Published:** 2022-12-22

**Authors:** Maria D. Gkioni, Konstantina Zeliou, Virginia D. Dimaki, Panayiotis Trigas, Fotini N. Lamari

**Affiliations:** 1 Laboratory of Pharmacognosy & Chemistry of Natural Products, Department of Pharmacy, School of Health Sciences, University of Patras, 265 04 Patras, Greece; 2 Laboratory of Systematic Botany, Department of Crop Science, School of Plant Sciences, Agricultural University of Athens, Iera Odos 75, 118 55 Athens, Greece

**Keywords:** sage, rosmarinic acid, carnosic acid, abietane diterpenes, volatiles, metabolomic profiling

## Abstract

*Salvia fruticosa* and *S. pomifera* subsp. *calycina* are native to Eastern Mediterranean and *S. pomifera* subsp. *pomifera* is endemic to Greece. The primary aim of this study was to develop an analytical methodology for metabolomic profiling and to study their efficacy in combating glycation, the major biochemical complication of diabetes. After sequential ultrasound-assisted extraction of 2 g of leaves with petroleum ether and 70% methanol, the volatile metabolites in the petroleum ether extracts were studied with GC-MS (Gas Chromatography-Mass Spectrometry), whereas the polar metabolites in the hydroalcoholic extracts were determined and quantified by UHPLC-DAD–ESI-MS (Ultra-High Performance Liquid Chromatography–Diode Array Detector–Mass Spectrometry). This methodology was applied to five populations belonging to the three native taxa. 1,8-Cineole was the predominant volatile (34.8–39.0%) in *S. fruticosa*, while *S. pomifera* had a greater content of α-thujone (19.7–41.0%) and β-thujone (6.0–39.1%). Principal Component Analysis (PCA) analysis of the volatiles could discriminate the different taxa. UHPLC-DAD-ESI-MS demonstrated the presence of 50 compounds, twenty of which were quantified. PCA revealed that not only the taxa but also the populations of *S. pomifera* subsp. *pomifera* could be differentiated. All *Salvia* samples inhibited advanced glycation end-product formation in a bovine serum albumin/2-deoxyribose assay; rosmarinic and carnosic acid shared this activity. This study demonstrates the antiglycation activity of *S. fruticosa* and *S. pomifera* extracts for the first time and presents a miniaturized methodology for their metabolomic profiling, which could aid chemotaxonomic studies and serve as a tool for their authentication and quality control.

## 1. Introduction

*Salvia* L. (Lamiaceae) includes approximately 980 species that are distributed almost worldwide [1]. A few of these are aromatic species that are used as flavoring agents and spices, but also as medicinal herbal products with commercial value. More than 100 volatiles have been found in the essential oil of the studied *Salvia* species, belonging to the classes of monoterpenes, sesquiterpenes, diterpenes, and non-isoprenoid compounds, usually with thujone, camphor, and 1,8-cineole as the most dominant ones [2]. Regarding non-volatiles, about 160 polyphenolic compounds have been identified from sage plants: flavonoids and their glycosides, anthocyanins, and phenolic acids with characteristic caffeic acid derivatives, such as rosmarinic acid, and phenolic diterpenes such as carnosic acid [3,4]. Most studies on the phytochemistry of *Salvia* taxa have focused on essential oils and have demonstrated that the chemical composition of essential oils varies greatly not only among different taxa but also within the same taxon [5,6,7,8].

Twenty-four *Salvia* taxa grow in the wild in Greece. *Salvia officinalis* L. (common sage) is limited to the northern part of the mainland and the Ionian islands. Three other *Salvia* taxa have historically and traditionally been viewed the same as the common sage in terms of the uses or even confused with that (the name “eleliphascos” was used for all of them) although a detailed analysis revealed certain differences [9]. Those taxa are: (1) *Salvia fruticosa* Mill. (Greek sage) distributed from Italy to West Syria, common almost throughout Greece, (2) *Salvia pomifera* L. subsp. *pomifera* endemic to Crete, Kithira, and Antikythira islands, and (3) *Salvia pomifera* subsp. *calycina* (Sm.) Hayek. (apple sage) distributed in southern Greece, growing also locally in West Anatolia [10]. Studies of their phytochemistry have shown that all these taxa share common compounds with *S. officinalis*, but also present a great chemodiversity, even within subspecies, which accounts for the difference in certain medicinal uses [4,6,7,9,11]. Most of the studies have evaluated essential oil composition, only a few evaluated polar secondary metabolites [11,12,13,14], and even fewer studies have measured both volatile and polar metabolites in the same plant material [15]. A thorough recording of the secondary metabolome among sage taxa may not only resolve chemotaxonomic issues but also guide authentication and quality control studies. In addition, analysis of their secondary metabolites may reveal the presence of new natural products and aid in the selection of superior genotypes of those medicinal plants in breeding efforts.

Non-clinical data have shown that *S. fruticosa* extracts have antioxidant, anti-inflammatory, antimicrobial, antiviral, spasmolytic, antihypertensive, estrogenic activity, anti-ulcer, and central nervous system effects [8,16,17,18,19]. Far fewer studies are focused on the biological properties of *S. pomifera* [13,14,19,20,21], and, in particular, on the antioxidant, antimicrobial, and cytotoxic properties.

Advanced glycation end-products (AGEs) are a heterogeneous class of covalently modified compounds that occur when reducing sugars or their metabolic media attack various substrates such as nucleic acids and phospholipids but primarily proteins. A series of those not completely defined non-enzymatic reactions, namely glycation, includes oxidative and non-oxidative pathways, which result in the formation of AGEs [22]. Thus, the association of AGEs with the development of diabetes and its complications (cataract, cardiomyopathy, neuropathy, retinopathy, and nephropathy) is crucial. Recently, plant extracts (of *S. officinalis* among them), and certain phenolic compounds have been evaluated for their effects on the formation of AGEs, presenting important anti-glycation and antioxidant effects [23,24]. However, no studies have been conducted on the efficacy of *S. fruticosa* and *S. pomifera*.

Since limited information is available on the chemodiversity of the Greek and apple sage, we embarked on the investigation of the chemical composition of the polar and non-polar extracts of three *S. pomifera* and two *S. fruticosa* wild populations from Greece by combining GC-MS and LC-MS techniques. We used an ultrasound-assisted extraction method that allowed the simultaneous analysis of both volatile and non-volatile compounds using only a few grams of plant material. Multivariate approaches were adopted to test if and how the populations and taxa differed. Furthermore, this study examined the ability of the hydroalcoholic extracts to inhibit AGEs formation, which has never been studied earlier.

## 2. Results and Discussion

Leaves from five populations from three *Salvia* taxa were collected and analyzed (two biological samples/population). In detail, two of *Salvia fruticosa* Mill. (from Fournoi island (North Aegean) and Rodini (Achaia, Peloponnese), mentioned as SF-S and SF-A, respectively), two of *Salvia pomifera* L. subsp. *pomifera* (from Elafonisi area and Sfakiano gorge (Crete), mentioned as SPP-E and SPP-FS, respectively) and one population of *Salvia pomifera* subsp. *calycina* (Sm.) Hayek from Parnitha Mountain in Attica (SPC-A).

### 2.1. GC-MS Analysis of Petroleum Ether Extracts for Volatile Profiling

The average petroleum ether (PE) extract yield was 8.96%, 4.15%, 12.76%, 7.40%, and 5.17% (*v*/*w*) for SPC-P, SPP-E, SPP-FS, SF-S, and SF-A, respectively. High yield values are explained by the fact that such organic solvents have the capacity to extract lipophilic compounds such as fatty acids and aliphatic hydrocarbons along with essential oil ingredients. Literature on PE extraction from *Salvia* species is limited; however, our results agree with those of Velickovic et al. [25] for other sage taxa (4.9 and 2.7%).

In total, forty-one (41) volatiles were identified in the PE extracts of the five examined *Salvia* populations, and fifteen were common in all samples (Table 1). Representative chromatograms are provided in Supplementary Information (Figures S1–S3). The total identified compounds ranged from 87 to 93% of the total peak area and were classified as monoterpenes and sesquiterpenes. Oxygenated monoterpenes and sesquiterpenes dominated every *Salvia* PE extract (Table 1).

The composition of volatiles in the *S. pomifera* subsp. *pomifera* populations were quite distinct from that in the *S. pomifera* subsp. *calycina* population, but thujones were the dominant ingredients in both taxa. Specifically, the main compounds in *S. pomifera* subsp. *calycina* (SPC-P) extracts were α-thujone and cubebol (approximately 20% and 10%, respectively). Additionally, β-thujone, (E)-caryophyllene, and myrcene were found in percentages of over 4.5%. The high values of cubebol and myrcene are in accordance with previous studies on the taxon [7,26]. On the other side, the main ingredients of the *S. pomifera* subsp. *pomifera* extracts were α-thujone (>25%) and β-thujone (>20%). In both *S. pomifera* subsp. *pomifera* extracts, (E)-caryophyllene was present at relatively high concentrations (>7.5%), whereas camphor was present at low concentrations (<1%) in agreement with the results of Karousou et al. [27]. Furthermore, the predominant compound in SPP-E was α-thujone whereas SPP-FS was dominated by β-thujone.

The most abundant compounds in *S. fruticosa* extracts were 1,8-cineole (>34%) and camphor (>5%) in accordance with previous studies [6,28,29]. Clearly, the two *S. fruticosa* populations belonged to the same chemotype.

### 2.2. LC Profiling and Determination of Polar Phenolic and Diterpene Metabolites in Hydroalcoholic Extracts

The yield of hydroalcoholic extracts of the *Salvia* populations ranged from 8.82 to 10.96%. The metabolites were analyzed and identified by UHPLC-DAD-ESI-MS analysis using both positive and negative ionization modes. Representative chromatograms are presented in Supplementary Information (Figures S4–S6). Table 2 illustrates the characterization of the 50 compounds that were detected in the hydroalcoholic *Salvia* extracts, and Table 3 presents the quantification results. The linearity calibration curves are shown in Figure S7.

The constituents are members of various classes of the polyphenolic spectrum, such as caffeic acid derivatives, flavonoid glycosides, flavonoid aglycones, and abietane diterpenes. Of the 50 compounds, 14 were common in all *Salvia* populations (Table 2). *S. fruticosa* populations showed richer chemical profiles than the *S. pomifera* ones. In all five *Salvia* populations, luteolin-7-*O*-glucoside (**12**) and luteolin glucuronide (**13**) were present, whereas the luteolin glycosides **10** and **11** were present only in *S. fruticosa*. Luteolin-7-*O*-glucoside was quantified in SPC-P and SPP-E populations and ranged between 0.66–1.11 mg/g DW, while luteolin glucuronide was also quantified in SF-S and SF-A and showed a variance of 0.41–2.16 mg/g DW. The flavonoids hispidulin (**31**), cirsimaritin or salvianolic acid F (**34**), and salvigenin (**42**) were found in all five populations but only compound **34** was quantifiable in everyone (0.19–0.67 mg/g DW). Apigenin and hispidulin glucosides (compounds **2**, **5**, **17**, **20**, **21**, **26**) were present in the *S. fruticosa* populations (Table 2).

Rosmarinic acid, the main metabolite in all populations, ranged from 3.80 to 12.55 mg/g DW (Table 3); the SPC-P population showed the highest value of rosmarinic acid (12.55 mg/g DW), while the two *S. pomifera* subsp. *pomifera* populations, the lowest values (<4 mg/g DW). *S. fruticosa* populations SF-S and SF-A contained 7.22 and 11.02 mg/g DW rosmarinic acid. Our results are in accordance with previous studies in *S. pomifera* and *S. fruticosa* samples [12]; in that study, rosmarinic acid in 15 *S. fruticosa* populations ranged from 1.00 to 10.72 mg/g DW and in two *S. pomifera* populations 2.46–6.74 mg/g DW. Vergine et al. [15] quantified 6.55 mg/g DW of rosmarinic acid in *S. fruticosa*. Sarrou et al. [11] reported a decrease in rosmarinic acid in *S. fruticosa* from 35.354 mg/g DW to 26.355 mg/g DW from August to September.

Salvianolic acid K (compound **25**) was also detected in all *Salvia* populations but was quantifiable only in SF ones (2.29–2.44 mg/g DW). Cvetkovikj et al. [12] reported the presence of <1.00–1.31 mg/g DW salvianolic acid K in 15 *S. fruticosa* populations.

Additionally, two unidentified compounds with MW of 598 (compound **9**) and 778 (compound **19**) were found in the *S. pomifera* populations, but to our knowledge, no study has reported any information about them. Both **9** and **19** were quantified only in the SPC-P and SPP-E populations in a range of concentrations of 2.36–0.82 mg/g DW and 1.32–1.74 mg/g DW, respectively.

Seven abietane diterpenes were present in the two *S. fruticosa* populations, specifically two rosmanol isomers (**33** and **35**), methoxy rosmanol or methoxycarnosol (**44**), two carnosol isomers (**45**) and (**46**), carnosic acid (**48**), and 12-*O*-methylcarnosic acid (**49**). In the mass spectrum of rosmanol (**33**), the fragment *m*/*z* 301 indicates decarboxylation [M-COO-H]^−^ [38]. Decarboxylation-derived fragments were also present in mass spectra of carnosol (*m*/*z* 285) and carnosic acid (*m*/*z* 287). Additionally, rosmanol displayed the fragment *m*/*z* 283, which corresponds to a further loss of water [38]. Unknown compound **39**, with an MW of 362, is possibly an abietane diterpene as well, due to the presence of the fragment *m*/*z* 287. Carnosic acid was present only in *S. fruticosa* populations at 6.35 and 7.22 mg/g DW. Carnosol (**45**) ranged between 7.51 and 9.11 mg/g DW in *S. fruticosa* populations. Those findings agree with previous literature where the same compound displayed 6.18 mg/g DW in an *S. fruticosa* population [12]. Moreover, an unidentified diterpene **40** was found in the *S. pomifera* populations as described in the study by Koutsoulas et al. [14] along with compound **43**. Both **40** and **43** were detected in quantifiable amounts in all *S. pomifera* populations and ranged between 3.24–4.83 mg/g DW and 5.06–16.38 mg/g DW, respectively. Compound **49** was present in the *S. pomifera* populations as previously mentioned [14]; SPP-E and SPP-FS contained approximately 5 mg/g DW of compound **49**.

### 2.3. Multivariate Analysis

**Figure 1:**
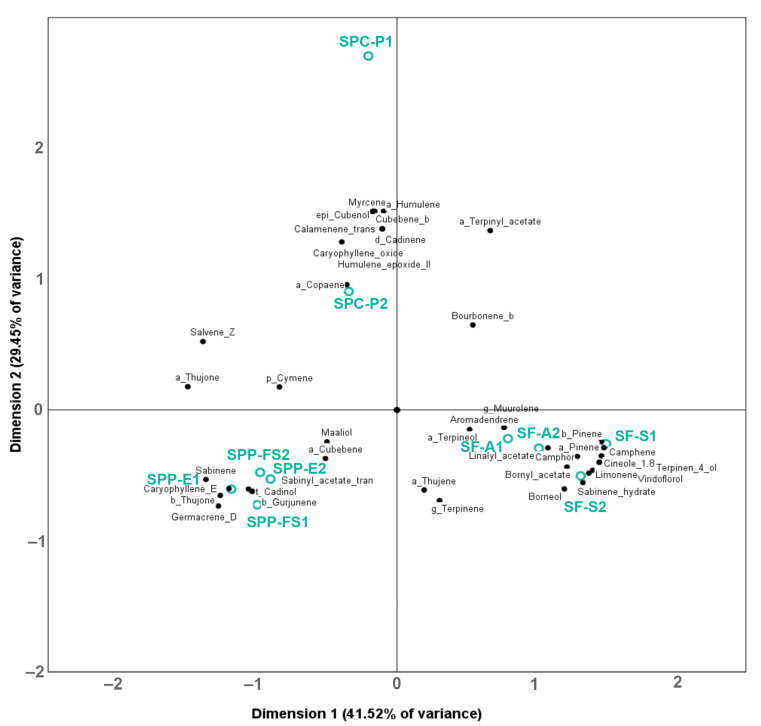
PCA biplots (after Varimax rotation) of volatile secondary metabolites in PE extracts of *Salvia* populations.

**Figure 2:**
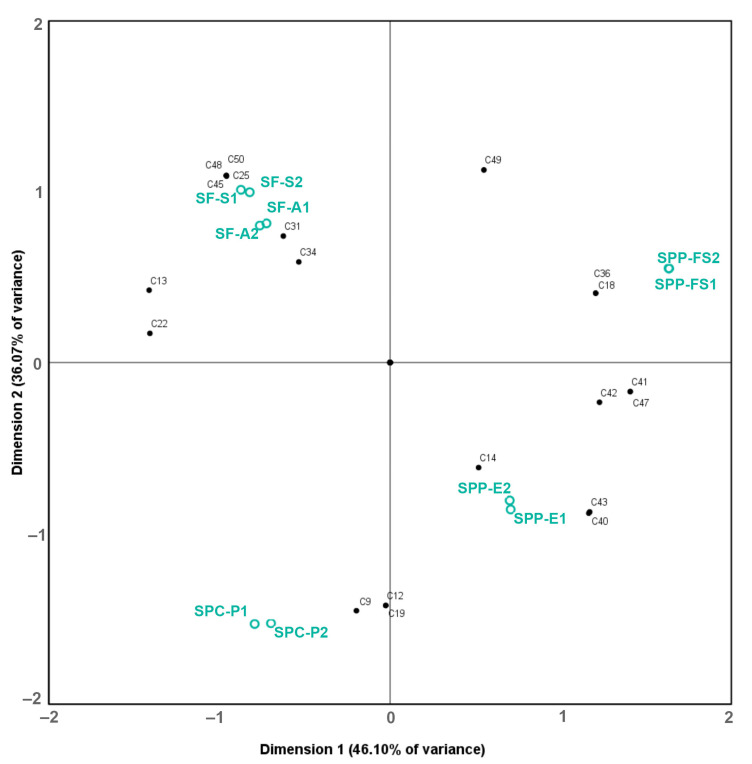
PCA biplots (after Varimax rotation) of polar secondary metabolites in hydroalcoholic extracts of *Salvia* populations. The numbering of compounds is in accordance with that in Table 2.

In order to test the power of this analytical toolkit to characterize the *Salvia* taxa and populations, biplots were produced to determine the association of the volatile and non–volatile chemical compounds with the samples (Figure 1 and Figure 2, respectively).

The % peak areas of 41 volatile compounds were subjected to PCA (Figure 1), and 70.97% of the total variability was explained by the first two principal components. PC 1 and PC 2 explained 41.52% and 29.45% of the variance, respectively. Based on the biplot analysis, all *S. pomifera* samples are associated mainly with α–thujone, (*Z*)–salvene, and sabinene; all *S. pomifera* ssp. *pomifera* samples are grouped closely together and are associated with germacrene D, β–thujone and (E)–caryophyllene, while *S. pomifera* ssp. *calycina* samples are associated with myrcene, β–cubebene, α–humulene, epi–cubenol and cubenol. Finally, 1,8–cineole, terpinene–4–ol, α–pinene, camphene, β–pinene, limonene, camphor, and sabinene–hydrate were associated with *S. fruticosa* samples.

The concentrations of the 20 most abundant ingredients of the polar extract were also subjected to PCA, and 82.17% of the total variability of the data was explained by the first three components (Figure 2); PC 1 and PC 2 explained 46.10% and 36. 07%, respectively. Based on the biplot analysis, all *S. fruticosa* samples were associated with salvionic acid K (**25**), carnosol (**45**), carnosic acid (**48**), and compound **50** (abietane diterpene); *S. pomifera* ssp. *pomifera* samples were not grouped together, but were associated with salvigenin (**42**), salviol (**47**), and compound **41** (7–methoxy rosmanol or methoxycarnosol). In addition, SPP-E and SPC-P samples were associated with compounds **9** and **19** and luteolin–7–*O*–glucoside (**12**); however, *S. pomifera* ssp. *calycina* samples were not closely associated with any of the quantified non-volatile ingredients. It is worth mentioning that the two SF samples in our study were collected at different times (SF-S in July and SF-A in September), but they were clustered together in both Figure 1 and Figure 2, showing that time-dependent changes in the concentration of the metabolites that may exist do not affect the discrimination from the other taxa.

### 2.4. Antiglycation Studies

First and foremost, the optimization of the method was performed. According to Maietta et al. [39], fluorescence monitoring with the 335/420 pair gives information on pentosidine-like AGEs, while the 370/440 pair, on vesperlysine-like AGEs. 2-Deoxy-d-ribose was chosen due to its higher glycation capacity as described by Monnier [40]. Trichloroacetic acid (TCA) was used to precipitate proteins and remove contaminants from the solution. Matsuura et al. [41] revealed that the use of 10% TCA did not affect AGEs structure. Aminoguanidine at the concentration of 7.5 mM showed 88.3 ± 2.2% and 86.4 ± 2.9% inhibition of pentosidine-like and vesperlysine-like AGE formation, respectively; those values agree with the literature since its reported IC50 is 5 ± 3 mM [42].

**Figure 3:**
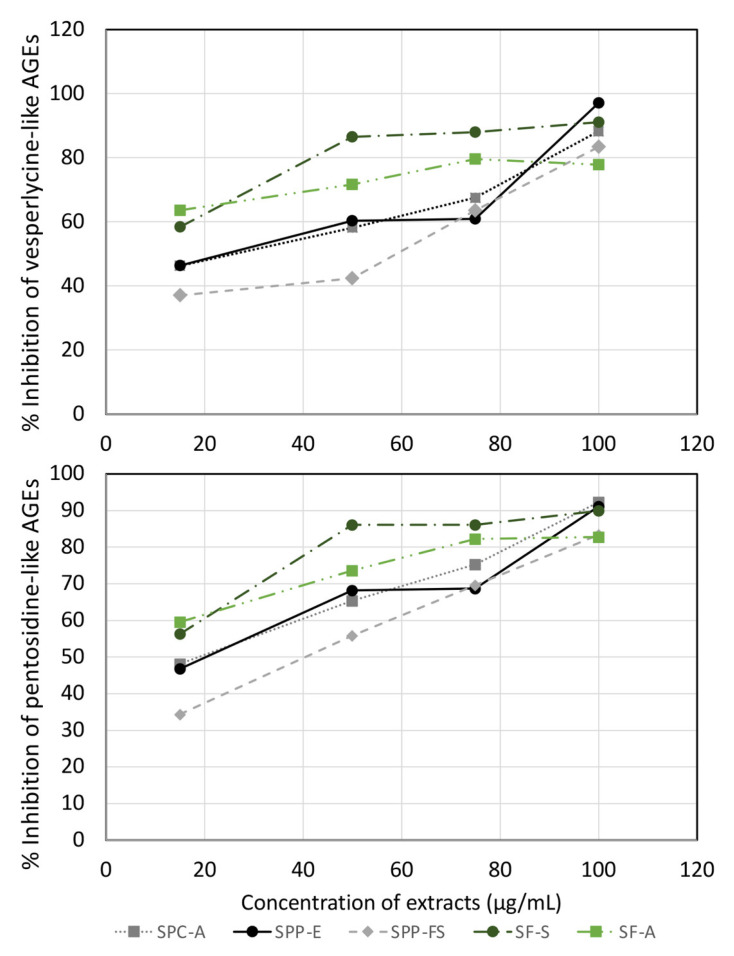
The % inhibition of pentosidine-like and vesperlycine-like AGEs by the hydroalcoholic extracts of *Salvia* leaf samples in a concentration range of 15 to 100 μg/mL.

The dose–response curves showing the % inhibition of vesperlysine-like and pentosidine-like AGEs formation by extracts in the concentration range of 15 to 100 μg/mL are presented in Figure 3. At the concentration of 100 μg/mL, all *Salvia* extracts showed strong inhibitory activity against AGEs formation (>77.9%). At lower concentrations, SF extracts scored higher inhibitory action than the *S. pomifera* ones. This finding might be due to the fact that SF populations contained carnosic acid (not present in *S. pomifera* ones) and high values of rosmarinic acid.

**Figure 4:**
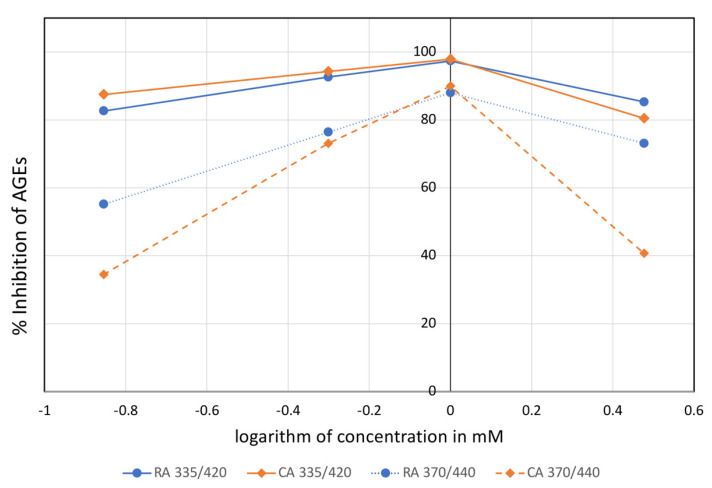
The % inhibition of pentosidine-like (370/440) and vesperlycine-like (335/420) AGEs by rosmarinic acid (RA) and carnosic acid (CA) in a concentration range of 0.14 to 3.00 mM.

Previous studies have revealed the antiglycation activity of those compounds, and we confirmed this in the current experimental set-up (Figure 4) [43,44]. Previously, rosmarinic acid and carnosic acid inhibited the formation of AGEs, carboxymethyl lysine and carboxyethyl lysine production in the BSA-glucose, BSA/glyoxal, and BSA/methylglyoxal assay systems, and significantly decreased the concentration of methylglyoxal and protein carbonylation [44].

In our BSA/2-deoxyribose assay system, both compounds showed nearly 100% and 90% inhibition of pentosidine-like and vesperlysine-like AGEs formation at the concentration of 1 mM, respectively. At a higher concentration, they seem to be less effective, but this apparent increase in AGEs production might be due to the quenching effect of the fluorescence of the tested compounds at higher concentrations, which has been repeatedly reported for natural products [42]. Notwithstanding, the beneficial inhibitory activity of AGEs formation has been reported for other sage ingredients, e.g., for caffeic acid derivatives and luteolin glycosides [24,43], as well; thus, the other ingredients might also contribute to the activity of the extracts.

## 3. Materials and Methods

### 3.1. Chemicals and Reagents

All solvents used for UHPLC-DAD-ESI-MS, i.e., acetonitrile (99.9%, LC/MS grade) and water (LC/MS grade), were purchased from Thermo Fisher Scientific (Waltham, MA, USA). Petroleum ether (95% GC/MS grade, 40–65 °C) and *n*-alkanes (C_8_–C_20_) were purchased from Carlo Erba Reagents S.A.S. (Barcelona, Spain) and Fluka, Sigma-Aldrich (Burlington, MA, USA), respectively. Methanol (99.8%, HPLC grade) and water (HPLC grade) used for the extraction were purchased from Thermo Fisher Scientific (Waltham, MA, USA). HPLC reference standards rosmarinic (≥99% analytical standard) and carnosic acid (≥90%) were purchased from Extrasynthese (Genay Cedex, France), while luteolin 7-*O*-glucoside (≥95%) was from Phytolab (Vestenbergsgreuth, Germany). Bovine Serum Albumin (BSA) Fraction V (lyophilized powder >95%) was purchased from Pan Biotech (Aidenbach, Germany), 2-deoxy-d-ribose (>99%) from Alfa Aesar (Kandel, Germany), aminoguanidine bicarbonate salt (>98%) from Sigma-Aldrich (St. Louis, MO, USA), sodium azide (>99%) and trichloroacetic acid (TCA) (>99.5%, pharma grade) from PanReac AppliChem ITW Reagents (Barcelona, Spain). Anhydrous sodium sulphate (>99%) was purchased from Penta Chemicals (Prague, Czech Republic) while disodium hydrogen phosphate (>99%) and sodium dihydrogen phosphate dihydrate (>99%) were purchased from Merck (Kenilworth, NJ, USA).

### 3.2. Plant Material

The aerial parts of five native Greek sage populations were collected on July of 2018 except SF-A that was collected in September of the same year. Plant material of SPC-P, SPP-E, and SPP-FS was collected and identified by Prof. Dr. P. Trigas, while their vouchers (ACA 6459, ACA 6433, ACA 6457) were deposited at the Agricultural University of Athens Herbarium (ACA). SF-S and SF-A were collected by K. Zeliou and M. D. Gkioni, respectively, and identified by the UPA Herbarium staff, where vouchers were deposited (UPA 22928, UPA 22929). Plant material was dried at room temperature in the dark and stored until extraction.

### 3.3. Extraction

For the extraction, previously described protocols were modified [12,25]. Two grams of dried leaves were grounded in the presence of liquid nitrogen and subsequently extracted with 20 mL petroleum ether (three times) and 20 mL 70% (aq.) methanol (three times) for 20 min each time in an ultrasonic bath (120 W, 40 kHz) at a temperature under 40 °C. Two biological replicates per population were extracted. The petroleum ether (PE) extracts obtained were condensed, dried over anhydrous sodium sulfate, and stored at −20 °C. The hydroalcoholic extracts obtained were freeze-dried (Freezone 6, Labconco, MO, USA) and stored at −20 °C.

### 3.4. Gas Chromatography-Mass Spectrometry (GC-MS)

The PE extracts were analyzed by GC-MS on a non-polar HP-5MS (30 m × 0.25 mm × 0.25 μm film thickness) column, using a 6890N GC interfaced with an 5975B mass spectrometer (Agilent Technologies Inc., Santa Clara, CA, USA) using electron impact (70 eV) ionization mode. Helium was used as the carrier gas; the flow rate was 1 mL/min and the injected volume was 1 μL in the splitless mode. Injection temperature was set at 280 °C, and the ion source was heated to 230 °C. The oven temperature program was 59 °C for 1 min, 59–66 °C (1 °C/min), 66–70 °C (1 °C/min), 70–110 °C (2 °C/min), 110–140 °C (1 °C/min), and 140–300 °C (30 °C/min). The relative content of each compound was calculated as the percentage of the peak area (peaks up to 70 min) to the total chromatographic peak area, and the results were expressed as means of two replicates for each population. The identification of the compounds was based on a comparison of their retention indices (RIs) relative to *n*-alkanes (C_8_–C_20_) and their spectra with those of the NIST Chemistry WebBook, SRD 69. The software used was AMDIS (Automated Mass spectral Deconvolution & Identification System v.2.73, NIST Institute) and Wsearch VS2020 (Wsearch Software by Frank Antolasic).

### 3.5. Ultra-High Performance Liquid Chromatography–Diode Array Detector–Mass Spectrometry (UHPLC-DAD-ESI-MS)

Chromatographic analyses of hydroalcoholic extracts were carried out using a Dionex UltiMate 3000 LC system (Thermo Fisher Scientific, Waltham, MA, USA) apparatus coupled to a quadrupole ion-trap Bruker amaZon SL MS equipped with an ESI interface (Bruker Daltonics, Billerica, MA, USA) at the Central Instrumental Analysis Laboratory of the University of Patras. UV–Vis detection was set at 254, 280, 330, and 380 nm and performed with a Dionex Ultimate DAD detector. The concentration of the samples was adjusted to 0.3 mg/mL in 25% *v*/*v* methanol. The separation was performed using an Acclaim 120 C18 (2.1 mm × 100 mm, 3 μm) (Thermo Fisher Scientific, Waltham, MA, USA). The flow rate was 0.3 mL/min, and the injection volume was 7 μL. The mobile phase consisted of 0.2% (*v*/*v*) formic acid in water (A) and 0.2% (*v*/*v*) formic acid in acetonitrile (B). The gradient elution started with 12% B and reached 19% at 5 min, 21% at 15 min, 30% at 18 min, 90% at 30 min, and 100% from 31–36 min. The column temperature was kept at 35 °C. The Bruker Compass DataAnalysis V4.2 software (Bruker Daltonics) was used for data processing. The identification was performed via the comparison of the elution order on C18 columns, UV–vis, and MS spectra with previous literature on sage and rosemary samples [12,14,30,31,32,33,34,35,36,37]. It is worth mentioning that rosemary (*Rosmarinus officinali*s L., 1753 or *Salvia rosmarinus* (L.) Schleid., 1852) is quite like sage in the composition of polyphenols mainly in terms of carnosic acid-derived diterpenes. Since reference compounds were not available for all the metabolites, we performed the quantification using one commercially available reference standard for each compound category as previously performed [30,31]. Luteolin 7-*O*-glucoside (at 330 nm), rosmarinic acid (at 280 nm), and carnosic acid (at 280 nm) were used for the quantification of flavonols, phenolic acids, and diterpenes, respectively (for more details, see Table 2). The standard curves that came up were y = 27.331x + 0.2094, R^2^ = 0.9993 for luteolin-7-*O* glucoside, y = 14.085x + 16.718, R^2^ = 0.9987 for rosmarinic acid, and y = 2.828x − 13.714, R^2^ = 0.9988 for carnosic acid. The chosen concentrations for luteolin-7-*O*-glucoside, rosmarinic acid, and carnosic acid were 2, 4, 7, 12, 25, 50, 60 μg/mL, 5, 10, 25, 50, and 100 μg/mL and 4.5, 9, 22.5, 45, and 90 μg/mL, respectively. Results are expressed as in mg g^−1^ dry extract weight.

### 3.6. Anti-Glycation Activity Determination Assay

The method was based on previous studies with slight modifications [39,41]. Bovine Serum Albumin (BSA) (0.625 mg/mL) was mixed with 2-deoxy-d-ribose (25 mM) and sodium azide (0.75 mM) in the presence or absence of inhibitors and samples. All solutions were prepared in phosphate buffer (pH 7.4; 0.1 M). Sage hydroalcoholic extracts were prepared at various concentrations (15–100 μg/mL), while aminoguanidine at 7.5 mM was used as a standard inhibitor. Rosmarinic and carnosic acid were examined in a concentration range of 0.14–3.00 mM. Samples were incubated at 37 °C in the dark for 6 days. At the end of the incubation, 10% *w*/*v* TCA was added, and the samples were centrifuged. Sediments were redissolved in the phosphate buffer, and fluorescence intensity was measured. The excitation/emission wavelength pairs were 335/420 nm and 370/440 nm.

### 3.7. Multivariate Analysis

The quantified volatile components, as well as the main polar metabolites of the hydroalcoholic extracts, were analyzed using principal component analysis (PCA) (followed by Varimax rotation with Kaiser normalization) with SPSS version 25.0 (IBM Corp., Armonk, NY, USA). The variables were standardized for a normalized PCA; the value of 0.001 was used for compounds that were not detected. For the volatile compounds, a matrix was created with 41 variables × 10 samples = 410 data points, while for methanolic extracts, the matrix was generated from 20 variables × 10 samples = 200 data points. Each component value of the Loading Plot graph was calculated in relation to the other, adjusting each value for the mean of each extraction. Biplots were produced with CATPCA (IBM Corp., Armonk, NY, USA).

## 4. Conclusions

With a miniaturized extraction approach of the plant material, we could monitor simultaneously the volatile and polar metabolites of *Salvia* leaves with only 2 g of starting material. Thus, thorough monitoring of the chemovariability of five populations of the three taxa was possible for the first time. A virtue of this approach is that it may facilitate studies on rare endemics and range-restricted sage species. The non-polar leaf extracts of *S. pomifera* populations had a high content of α-thujone (19.7–41.0%) and β-thujone (6.0–39.1%), while *S. fruticosa* extracts exhibited a greater content of 1,8-cineole (34.8–39.0%). UPLC-DAD-ESI-MS demonstrated the presence of a total of 50 compounds; 14 were present in all studied populations. Carnosic acid was determined only in *S. fruticosa* (6.35–7.22 mg/g DW), while rosmarinic acid concentration ranged from 3.80 to 12.55 mg/g DW; the lowest values were noted in SPP ones. Multivariate analysis showed that this analytical methodology can discriminate the three *Salvia* taxa and can be used in chemotaxonomic and authentication efforts with larger numbers of populations. For the first time, we show the dose-dependent high antiglycation activity of the polar extracts of those three taxa and confirm in our experimental set-up (BSA/2-deoxy ribose) previous studies showing the antiglycation activity of rosmarinic and carnosic acid, showing that those two compounds contribute greatly to the antiglycation activity of the extracts. In summary, the secondary metabolome of Greek and Cretan sage populations was recorded with the usage of a strategy requiring minute amounts of plant material. The promising anti-glycation activities of the methanolic extracts of the leaves are presented.

## Figures and Tables

**Table 1:** The major volatile compounds in petroleum ether extracts of the leaves of the five *Salvia* populations using GC/MS. The results are expressed as a percentage of the total peak area (means ± standard deviation), while the limit for identification and quantification was set at 0.10%.

Peak No.	Compound	RI _(th.)_	RI _(cal.)_	SPC-P	SPP-E	SPP-FS	SF-S	SF-A
V1	(Z)-Salvene	847	843	0.50 ± 0.03	0.42 ± 0.08	0.29 ± 0.01	n.d.	0.2 *
V2	α-Thujene	924	919	0.34 *	0.26 *	0.39 ± 0.04	0.43 ± 0.07	0.26 ± 0.01
V3	α-Pinene	932	923	0.89 ± 0.01	0.79 ± 0.46	0.70 ± 0.09	4.84 ± 0.05	4.29 ± 0.26
V4	Camphene	946	940	0.15 ± 0.01	n.d.	0.39 ± 0.38	4.53 ± 0.10	2.89 ± 0.25
V5	Sabinene	969	965	0.47 ± 0.03	1.75 ± 1.22	1.82 ± 0.19	n.d.	0.37 ± 0.08
V6	β-Pinene	974	966	0.79 ± 0.14	0.67 ± 0.3	0.55 ± 0.06	4.39 ± 0.49	5.12 ± 0.68
V7	Myrcene	988	987	5.00 ± 1.07	0.77 ± 0.03	0.87 ± 0.02	1.67 ± 0.61	1.25 ± 0.06
V8	p-Cymene	1020	1017	0.48 ± 0.05	0.60 ± 0.59	0.47 ± 0.26	0.49 *	0.34 *
V9	Limonene	1024	1019	0.53 *	0.62 ± 0.33	0.44 ± 0.17	1.44 ± 0.05	1.27 ± 0.16
V10	1,8-Cineole	1026	1021	1.39 *	3.43 ± 2.22	2.45 ± 0.18	34.76 ± 1.58	39.01 ± 1.15
V11	γ-Terpinene	1054	1047	0.14 *	0.42 ± 0.33	0.30 ± 0.14	0.45 ± 0.09	0.24 ± 0.01
V12	cis-Sabinene hydrate	1065	1058	n.d.	-	0.16 ± 0.04	0.28 ± 0.08	0.27 ± 0.08
V13	α-Thujone	1101	1099	19.65 ± 1.53	40.99 ± 9.2	25.84 ± 1.5	1.37 ± 0.12	1.34 ± 0.65
V14	β-Thujone	1112	1113	6.01 ± 0.34	21.36 ± 6.63	39.10 ± 5.40	2.88 ± 0.06	6.07 ± 6.30
V15	Camphor	1141	1137	0.22 *	1.04 ± 0.81	0.62 ± 0.53	11.20 ± 0.03	5.07 ± 5.99
V16	Borneol	1165	1164	n.d.	0.83 *	0.71 *	2.08 ± 0.02	0.67 ± 0.7
V17	Terpinen-4-ol	1174	1174	n.d.	n.d.	n.d.	0.22 ± 0.08	1.14 ± 1.37
V18	α-Terpineol	1186	1186	n.d.	n.d.	n.d.	n.d.	1.64 *
V19	Linalyl acetate	1254	1255	n.d.	n.d.	n.d.	0.68 ± 0.39	n.d.
V20	Bornyl acetate	1284	1278	n.d.	n.d.	0.28 *	1.12 ± 0.52	0.72 *
V21	trans-Sabinyl acetate	1289	1289	n.d.	0.26 ± 0.18	0.13 *	n.d.	n.d.
V22	α-Cubebene	1348	1339	n.d.	n.d.	0.20 *	n.d.	n.d.
V23	α-Terpinyl acetate	1346	1341	4.43 ± 0.76	0.26 *	0.21 *	0.51 ± 0.16	0.3 ± 0.17
V24	α-Copaene	1374	1363	2.34 ± 0.48	n.d.	3.33 *	0.18 *	n.d.
V25	β-Burbonene	1387	1368	0.24 *	n.d.	0.23 *	0.28 *	0.17 *
V26	β-Cubebene	1387	1378	0.27 ± 0.09	n.d.	n.d.	n.d.	n.d.
V27	(E)-Caryophyllene	1417	1404	4.89 ± 1.01	7.63 ± 0.82	7.84 ± 0.96	4.74 ± 0.78	3.08 ± 0.59
V28	β-Gurjunene	1433	1414	n.d.	0.96 *	0.65 ± 0.28	n.d.	n.d.
V29	Aromadendrene	1439	1423	n.d.	n.d.	n.d.	0.19 *	n.d.
V30	α-Humulene	1452	1439	2.39 ± 0.63	0.56 ± 0.12	0.44 ± 0.05	0.98 ± 0.31	0.42 ± 0.08
V31	γ-Muurolene	1478	1463	n.d.	n.d.	n.d.	0.24 *	n.d.
V32	Germacrene D	1484	1468	n.d.	0.39 ± 0.37	0.34 ± 0.12	n.d.	n.d.
V33	epi-Cubebol	1493	1481	0.71 ± 0.41	n.d.	n.d.	n.d.	n.d.
V34	Cubebol	1514	1502	10.24 ± 2.66	n.d.	n.d.	n.d.	n.d.
V35	trans-Calamene	1521	1509	0.87 *	n.d.	n.d.	n.d.	n.d.
V36	δ-Cadinene	1522	1511	0.61 *	n.d.	0.37 *	n.d.	n.d.
V37	Maaliol	1566	1548	n.d.	n.d.	0.53 *	n.d.	n.d.
V38	Caryophyllene oxide	1582	1564	1.07 ± 0.69	1.00 ± 0.49	1.07 ± 0.31	0.64 ± 0.63	0.57 ± 0.14
V39	Viridiflorol	1592	1574	n.d.	n.d.	0.26 *	0.46 ± 0.21	0.58 ± 0.03
V40	Humulene epoxide ΙΙ	1608	1591	0.59 *	n.d.	n.d.	n.d.	n.d.
V41	τ-Cadinol	1638	1604	n.d.	0.47 ± 0.23	0.42 *	n.d.	n.d.
Total identified%				87.08 ± 1.36	88.48 ± 10.16	93.07 ± 1.34	91.26 ± 0.09	87.46 ± 2.88
Number of identified compounds				26	22	31	26	25
Oxygenated%				43.91 ± 6.98	68.97 ± 11.67	70.26 ± 1.76	56.21 ± 2.48	57.43 ± 0.61
Hydrocarbons%				43.17 ± 5.61	19.52 ± 1.51	22.98 ± 2.59	35.06 ± 2.39	30.03 ± 2.27

* Compounds detected only in one of the two replicates. Abbreviations: RI _(th)_: theoretical retention index; RI _(calc)_: calculated retention index; n.d.: not detected.

**Table 2:** Polar metabolites identified in hydroalcoholic extracts of the five *Salvia* samples using UHPLC–DAD–ESI–MS on a C18 column. The retention times, the molecular weight, the ions observed after positive or negative ionization with a description of the ion origin with their relative abundance in parentheses, and the UV–vis absorption maxima are presented herein. The *Salvia* sample in which each compound was determined is presented in the eighth column. The previous studies that helped the characterization are provided in the last column.

Peak No.	Rt (min)	Tentative Identification	M.W.	Positive Ionization *m/z* (% Relative Intensity)	Negative Ionization *m/z* (% Relative Intensity)	λmax (nm)	Occurrence in Samples	References
C1	3.8	Coumaroyl-apiosyl-glucose	458	481 [M+Na]^+^ (100) 476 [M+NH_4_]^+^ (28)	457 [M-H]^−^ (100)	n.dtm.	SF-A	[30]
C2	4.3	Apigenin *O*-pentoside	402	425 [M+Na]^+^ (100) 420 [M+NH_4_]^+^ (39) 441 [M+K]^+^ (8)	401 [M-H]^−^ (100) 447 [M+FA-H]^−^ (47) 515 [M+TFA-H]^−^ (26)	n.dtm.	SPC-P, SF-S, SF-A	[31]
C3	4.5	Medioresinol	388	411 [M+Na]^+^ (100) 427 [M+K]^+^ (45) 406 [M+NH_4_]^+^ (21)	387 [M-H]^−^ (100)	216, 325	All	[32]
C4	4.7	Unknown	386	409 [M+Na]^+^ (100) 387 [M+H]^+^ (29)	431 [M+FA-H]^−^ (100) 499 [M+TFA-H]^−^ (21) 421 [M+Cl]^−^ (15)	n.dtm.	SPC-P, SPP-E, SPP-FS	
C5	4.7	Saponarin (Apigenin 6-C-glucoside-7-*O*-glucoside) or Apigenin 8-C-glucoside-7-*O*-glucoside	594	595 [M+H]^+^ (100) 617 [M+Na]^+^ (28)	593 [M-H]^−^ (100)	214, 272, 340	SF-S, SF-A	[12,30]
C6	5.8	1-O-Caffeoyl-β-D-apiofuranosyl-(1→6)-β-D-glucopyranoside	474	497 [M+Na]^+^ (100) 513 [M+K]^+^ (36)	519 [M+FA-H]^−^ (100)	n.dtm.	SF-S, SF-A	[33]
C7	6.5	6-Hydroxyluteolin 7-*O*-glucoside	464	465 [M+H]^+^ (100) 541 [M+2K+H]^+^ (79)	463 [M-H]^−^ (100)	217, 282, 344	SPC-P, SPP-E, SPP-FS#	[12]
C8	6.5	6-Hydroxyluteolin 7-*O*-glucuronide	478	479 [M+H]^+^ (100) 501 [M+Na]^+^ (21) 523 [M+2Na-H]^+^ (9) 517 [M+K]^+^ (8)	477 [M-H]^−^ (100) 499 [M+Na-2H]^−^ (20)	217, 283, 345	SPP-FS#, SF-S, SF-A	[12,30,31]
C9	7.7	Unknown	598	621 [M+Na]^+^ (100) 616 [M+NH_4_]^+^ (87) 599 [M+H]^+^ (85)	597 [M-H]^−^ (100) 619 [M+Na-2H]^−^ (23)	200, 218, 275	SPC-P, SPP-E, SPP-FS	
C10	7.8	Luteolin *O*-rutinoside isomer	594	595 [M+H]^+^ (100) 617 [M+Na]^+^ (19)	593 [M-H]^−^ (100)	219, 350	SF-S, SF-A	[12,30,31,34]
C11	8.1	Luteolin *O*-rutinoside isomer	594	595 [M+H]^+^ (100) 617 [M+Na]^+^ (17)	593 [M-H]^−^ (100) 615 [M+Na-2H]^−^ (21)	219, 350	SF-S, SF-A	[12,30,31,34]
C12	8.4	Cynaroside (Luteolin 7-*O*-glucoside) ^a^	448	449 [M+H]^+^ (100) 471 [M+Na]^+^ (11) 287 [M-glucoside+H]^+^ (8)	447 [M-H]^−^ (100) 561 [M+TFA-H]^−^ (12) 493 [M+FA-H]^−^ (6) 895 [2M-H]^−^ (6)	230, 268, 348	All	[12,14,30,31,32]
C13	8.6	Luteolin glucuronide	462	463 [M+H]^+^ (100) 485 [M+Na]^+^ (12) 507 [M+2Na-H]^+^ (3) 287 [M-glucuronide+H]^+^ (4)	461 [M-H]^−^ (100) 483 [M+Na-2H]^−^ (23) 923 [2M-H]^−^ (8)	217, 268, 347	All	[12,14,30,31,32,34]
C14	9.3	Nepitrin (6-Methoxyluteolin-7-glucoside) or Isorhamnetin-hexoside	478	479 [M+H]^+^ (100) 501 [M+Na]^+^ (34) 317 [M-glucoside+H]^+^ (15)	477 [M-H]^−^ (100) 591 [M+TFA-H]^−^ (11) 513 [M+Cl]^−^ (7)	218, 270, 346	All	[32,34]
C15	9.7	Salvianolic acid C	492	493 [M+H]^+^ (100) 515 [M+Na]^+^ (26)	491 [M-H]^−^ (100)	219, 272, 346	All	[34]
C16	10.5	Sagerinic acid	720	743 [M+Na]^+^ (100) 738 [M+NH_4_]^+^ (79)	719 [M-H]^−^ (100)	200, 280	SPC-P, SPP-FS, SF-S, SF-A	[14,31]
C17	11.0	Apigenin *O*-rutinoside	578	579 [M+H]^+^ (100)	577 [M-H]^−^ (100)	n.dtm.	SF-S, SF-A	[12,30]
C18	11.4	Salvianolic acid B	718	736 [M+NH_4_]^+^ (100) 743 [M+Na]^+^ (77) 757 [M+K]^+^ (16) 323 [M-DSS-DSS+H]^+^ (57) 521 [M-DSS+H]^+^ (38)	717 [M-H]^−^ (100) 739 [M+Na-2H]^−^ (27)	219, 285, 342	SPP-FS, SF-S, SF-A	[30,31,35]
C19	11.6	Unknown	778	779 [M+H]^+^ (100) 796 [M+NH_4_]^+^ (83) 801 [M+Na]^+^ (77) 409 [M+H+K]^2+^ (16)	777 [M-H]^−^ (100) 799 [M+Na-2H]^−^ (20)	231, 265, 274	SPC-P, SPP-E, SPP-FS	
C20	11.8	Hispidulin 7-O-rutinoside	608	609 [M+H]^+^ (100) 631 [M+Na]^+^ (24)	607 [M-H]^−^ (100)	n.dtm.	SF-S, SF-A	[12]
C21	12.1	Apigenin-glucuronide	446	447 [M+H]^+^ (100) 469 [M+Na]^+^ (21) 271 [M-glucuronic+H]^+^	445 [M-H]^−^ (100)	219, 268, 335	SPC-P, SF-S, SF-A	[12,30]
C22	12.5	Rosmarinic acid ^a^	360	383 [M+Na]^+^ (100) 163 [M-CA-H_2_O+H]^+^ (58) 361 [M+H]^+^ (13) 721[2M+H]^+^ (9)	359 [M-H]^−^ (100) 719 [2M-H]^−^ (29) 381 [M+Na-2H]^−^ (8)	222, 300sh, 330	All	[12,14,30,32,33,34,35]
C23	12.8	Luteolin glucuronide or hispidulin glucoside	462	463 [M+H]^+^ (100) 485 [M+Na]^+^ (30)	461 [M-H]^−^ (100)	219, 274, 335	All	[12,30,31,34]
C24	13.3	Hispidulin glucuronide	476	477 [M+H]^+^ (100) 499 [M+Na]^+^ (19) 301 [M-glucuronic+H]^+^ (17)	475 [M-H]^−^ (100)	n.dtm.	SPC-P, SPP-E, SPP-FS	[12,30,31]
C25	13.4	Salvianolic acid K	556	579 [M+Na]^+^ (100) 574 [M+NH_4_]^+^ (41) 556 [M+H]^+^ (32)	555 [M-H]^−^ (100) 577 [M+Na-2H]^−^ (15)	219, 289, 330	All	[12,30]
C26	13.7	Hispidulin glucuronide	476	477 [M+H]^+^ (100) 499 [M+Na]^+^ (29)	475 [M-H]^−^ (100) 577 [M+TFA-H]^−^ (9) 521 [M+FA-H]^−^ (9)	336	SF-S, SF-A	[12,30,31]
C27	14.0	Salvianolic acid C	492	493 [M+H]^+^ (100) 515 [M+Na]^+^ (17)	491 [M-H]^−^ (100)	222, 266, 296, 347	SPC-P, SPP-E, SPP-FS	[35]
C28	14.2	Hispidulin glucuronide	476	477 [M+H]^+^ (100) 499 [M+Na]^+^(13)	475 [M-H]^−^ (100)	330	SPC-P, SPP-E, SPP-FS	[12,30,31]
C29	14.2	Luteolin glucuronide or Hispidulin-glucoside	462	463 [M+H]^+^ (100) 485 [M+Na]^+^ (22)	461 [M-H]^−^ (100)	336	SF-S, SF-A	[12,30,31,34]
C30	20.1	Nepetin (6-Methoxyluteolin)	316	317 [M+H]^+^ (100)	315 [M-H]^−^ (100) 429 [M+TFA-H]^−^ (15)	330	SPP-E, SF-S, SF-A	[12,14]
C31	21.9	Hispidulin	300	301 [M+H]^+^ (100)	299 [M-H]^−^ (100)	221, 278, 340	All	[14,31,34]
C32	22.3	Cirsiliol	330	331 [M+H]^+^ (100)	329 [M-H]^−^ (100)	330	SPP-E, SF-S, SF-A	[12,32]
C33	23.4	Rosmanol	346	301 [Μ-46+H]^+^ (100) 369 [M+Na]^+^ (84) 347 [M+H]^+^ (28) 715 [2M+Na]^+^ (18) 283 [Μ-64+H]^+^ (14)	345 [M-H]^−^ (100) 691 [2M-H]^−^ (44) 459 [M+TFA-H]^−^ (25) 283 [Μ-COO-H_2_O-H]^−^ (11) 301 [Μ-COO-H]^−^ (8)	280	SF-S, SF-A	[12,14,30,34,36]
C34	23.7	Cirsimaritin or Salvianolic acid F	314	315 [M+H]^+^ (100) 337 [M+Na]^+^ (34)	313 [M-H]^−^ (100)	221, 280, 336	All	[12,14,34,35]
C35	24.0	Rosmanol isomer	346	369 [M+Na]^+^ (20)	345 [M-H]^−^ (100)	280, 330	SF-S, SF-A	[12,14,30,32,34];
C36	24.2	Rosmaridiphenol or Pomiferin F	316	317 [M+H]^+^ (100) 283 [Μ-34+H]^+^ (15)	315 [M-H]^−^ (100) 429 [Μ+TFA-H]^−^ (35)	280	All	[32]
C37	24.7	Unknown	332	355 [M+Na]^+^ (100) 333 [M+H]^+^ (50) 371 [M+K]^+^ (16)	331 [M-H]^−^ (100)	n.dtm.	All	
C38	24.9	Genkwanin	284	285 [M+H]^+^ (100)	283 [M-H]^−^ (100)	330	SPP-E, SPP-FS, SF-S, SF-A	[14,34]
C39	25.3	Abietane diterpene	362	385 [M+Na]^+^ (68) 380 [M+NH_4_]^+^ (25) 345 [Μ-18+H]^+^ (100)	361 [M-H]^−^ (100) 399 [M+K-2H]^−^ (100) 287 [Μ-74]^−^ (94)	280	SPC-P, SPP-E, SPP-FS	
C40	26.0	Abietane diterpene	318	317 [M+H]^+^ (15) 336 [M+NH_4_]^+^ (28) 659 [2M+Na]^+^ (22) 637 [2M+H]^+^ (21) 283 [Μ-36+H]^+^ (94) 301 [Μ-H_2_O+H]^+^ (100)	317 [M-H]^−^ (100) 363 [M+FA-H]^−^ (36) 635 [2M-H]^−^ (27)	284	SPC-P, SPP-E, SPP-FS	[14]
C41	26.4	7-Methoxy rosmanol	360	283 [M-78+H]^+^ (100) 383 [Μ+Na]^+^ (22) 721 [2M+H]^+^ (52)	405 [M-H]^−^ (100) 359 [M-H]^−^ (49)	281	SPP-E, SPP-FS	[36]
C42	26.5	Salvigenin	328	329 [M+H]^+^ (100) 351 [M+Na]^+^ (21) 679 [2M+Na]^+^ (13)		222, 276, 330	All	[34]
C43	26.7	2α-Hydroxy-*O*-methyl-pisiferic acid	346	710 [2M+NH_4_]^+^ (28) 715 [2M+Na]^+^ (21) 693 [2M+H]^+^ (13) 329 [Μ-18+H]^+^ (100) 283 [Μ-64+H]^+^ (41)	345 [M-H]^−^ (33) 691 [2M-H]^−^ (100) 301 [Μ-COO-H]^−^ (46)	207, 223sh, 284	SPC-P, SPP-E, SPP-FS	[37]
C44	27.0	7-Methoxy rosmanol or Methoxycarnosol	360	361 [M+H]^+^ (100) 383 [Μ+Na]^+^ (91) 743 [2M+Na]^+^ (20)	359 [M-H]^−^ (100)	n.dtm.	SF-S, SF-A	[32,36]
C45	27.2	Carnosol	330	331 [M+H]^+^ (100) 661 [2M+H]^+^ (51) 683 [2M+Na]^+^ (67) 353 [M+Na]^+^ (34) 285 [Μ-46+H]^+^ (20)	659 [2M-H]^−^ (100) 329 [M-H]^−^ (63) 285 [Μ-COO-H]^−^ (20)	219, 284	SPC-P, SPP-FS, SF-S, SF-A	[12,14,30,34,37]
C46	27.7	Carnosol isomer	330	331 [M+H]^+^ (100) 353 [M+Na]^+^ (92) 683 [2M+Na]^+^ (27) 285 [Μ-46+H]^+^ (10)	329 [M-H]^−^ (63) 285 [Μ-COO-H]^−^ (36)	n.dtm.	SF-S, SF-A	[12,14,30,34]
C47	28.3	Salviol	302	325 [Μ+Na]^+^ (38) 285 [Μ-H_2_O+H]^+^ (82)	603 [2M-H]^−^ (100) 649 [2M+FA-H]^−^ (56) 347 [Μ+FA-H]^−^ (57)	284	SPC-P, SPP-E, SPP-FS	[34,37]
C48	28.9	Carnosic acid ^a^	332	287 [Μ-46+H]^+^ (100) 687 [2M+Na]^+^ (78) 333 [M+H]^+^ (33) 355 [M+Na]^+^ (18)	331 [M-H]^−^ (100) 663 [2M-H]^−^ (34) 287 [Μ-COO-H]^−^ (5)	284	SF-S, SF-A	[12,14,30,34,36]
C49	29.7	12-Methylcarnosic acid	346	301 [M+H]^+^ (100) 347 [M+H]^+^ (8) 715 [2M+Na]^+^ (81) 369 [M+Na]^+^ (30)	345 [M-H]^−^ (100)	285	All	[14]
C50	29.9	Abietane diterpene	318	301 [Μ-H_2_O+H]^+^ (100) 659 [2M+Na]^+^ (37) 341 [M+Na]^+^ (24)	317 [M-H]^−^ (100) 635 [2M-H]^−^ (21)	285	SF-S, SF-A	[14]

^a^: A standard was used for the identification. Abbreviations: FA: formic acid, TFA: trifluoroacetic acid, DSS: dashensu, CA; carnosic acid; n.dtm.: not determined. # in the eighth column shows the sample in which the two compounds co-eluted.

**Table 3:** Concentration of the major polar metabolites in mg g^−1^ dry extract weight in each population. The first column shows the peak numbers as presented in Table 2 and the second column, the wavelength at which the quantification was performed.

Peak No.	UV (nm)	Compound	SPC-P	SPP-E	SPP-FS	SF-S	SF-A
			mg/g DW				
C9	280	Unknown ^b^	2.36 ± 0.08	0.82 ± 0.03	n.q.	n.d.	n.d.
C12	330	Luteolin 7-*O*-glucoside ^a^	0.66 ± 0.05	1.11 ± 0.08	n.q.	n.q.	n.q.
C13	330	Luteolin glucuronide ^a^	0.75 ± 0.04	0.41 ± 0.01	n.q.	2.16 ± 0.30	1.14 ± 0.02
C14	330	6-Methoxyluteolin-7-glucoside or Isorhamnetin-hexoside ^a^	n.q.	0.80 ± 0.02	n.q.	n.q.	n.q.
C18	280	Salvianolic acid B ^b^	n.d.	n.d.	1.09 ± 0.05	n.q.	n.q.
C19	280	Unknown ^a^	1.32 ± 0.07	1.74 ± 0.15	n.q	n.d.	n.d
C22	280	Rosmarinic acid ^b^	12.55 ± 0.41	3.80 ± 0.15	3.93 ± 0.31	7.22 ± 0.49	11.02 ± 0.33
C25	280	Salvianolic acid K ^b^	n.q.	n.q.	n.q.	2.44 ± 0.18	2.29 ± 0.02
C31	330	Hispidulin ^a^	n.q.	n.q.	n.q.	0.39 ± 0.01	n.q.
C34	330	Cirsimaritin or Salvianolic acid F ^a^	0.18 ± 0.09	0.67 ± 0.06	0.19 ± 0.02	0.64 ± 0.06	0.54 ± 0.03
C36	280	Rosmaridiphenol or Pomiferin F ^c^	n.q.	n.q.	3.11 ± 0.39	n.q.	n.q.
C40	280	Abietane diterpene ^c^	3.24 ± 0.12	4.78 ± 0.43	4.83 ± 0.37	n.d.	n.d.
C41	280	7-Methoxy rosmanol ^c^	n.d.	3.03 ± 0.22	2.23 ± 0.14	n.d.	n.d.
C42	330	Salvigenin ^b^	n.q.	3.17 ± 0.11	2.42 ± 0.23	1.43 ± 0.13	1.43 ± 0.21
C43	280	2α-hydroxy-*O*-methyl-pisiferic acid ^c^	5.06 ± 0.22	15.19 ± 0.45	16.38 ± 0.79	n.d.	n.d.
C45	280	Carnosol ^c^	n.q.	n.d.	n.q.	7.51 ± 0.21	9.11 ± 0.56
C47	280	Salviol ^c^	n.q.	10.11 ± 0.31	7.99 ± 0.42	n.d.	n.d.
C48	280	Carnosic acid ^c^	n.d.	n.d.	n.d.	7.22 ± 0.50	6.35 ± 0.24
C49	280	12-Methyl carnosic acid ^c^	n.q.	5.53 ± 0.44	5.21 ± 0.17	4.97 ± 0.49	5.80 ± 0.27
C50	280	Abietane diterpene ^c^	n.d.	n.d.	n.d.	4.51 ± 0.26	4.13 ± 0.23

The superscript letters indicate the standard that was used for the quantification, as follows: a: luteolin 7-*O*-glucoside, b: rosmarinic acid, c: carnosic acid. Abbreviations: n.d.: not detected; n.q.: not quantified.

## Data Availability

Data will be made available on request.

## Supplementary Materials

The following supporting information can be downloaded at: https://www.mdpi.com/article/10.3390/molecules28010093/s1. Figure S1: Representative total ion GC chromatogram of the petroleum ether extract of the leaves of the population SPC-A, Figure S2. Representative total ion GC chromatogram of the petroleum ether extract of the leaves of the population SPP-E, Figure S3. Representative total ion GC chromatogram of the petroleum ether extract of the leaves of the population SF-S, Figure S4. Representative chromatogram from the UHPLC-DAD-MS analysis of the hydroalcoholic extract of the leaves of the population SPC-A at 280 nm (1, black), at 330 nm (2, green), the total ion current after positive ionization (3, red) and negative ionization (4, blue), Figure S5. Representative chromatogram from the UHPLC-DAD-MS analysis of the hydroalcoholic extract of the leaves of the population SPP-FS at 280 nm (1, black), at 330 nm (2, green), the total ion current after positive ionization (3, red) and negative ionization (4, blue), Figure S6. Representative chromatogram from the UHPLC-DAD-MS analysis of the hydroalcoholic extract of the leaves of the population SF-S at 280 nm (1, black), at 330 nm (2, green), the total ion current after positive ionization (3, red) and negative ionization (4, blue), Figure S7. Linearity calibration curves of luteolin-7-*O*-glucoside, rosmarinic acid, and carnosic acid that were used as external standards in the UHPLC-DAD-MS analysis of the hydroalcoholic extracts of the leaves of the various Salvia taxa. Each one was used for a different category of compounds.

## Sample Availability

Samples are not available from the authors.

## References

[1] Will M., Claßen-Bockhoff R. (2017). Time to Split Salvia s.l. (Lamiaceae)—New Insights from Old World Salvia Phylogeny. Mol. Phylogenetics Evol..

[2] Fu Z., Wang H., Hu X., Sun Z., Han C. (2013). The Pharmacological Properties of Salvia Essential Oils. J. Appl. Pharm. Sci..

[3] Nikolova M., Aneva I., Georgiev V., Pavlov A. (2017). European Species of Genus Salvia: Distribution, Chemodiversity and Biological Activity. Salvia Biotechnology.

[4] Wu Y.-B., Ni Z.-Y., Shi Q.-W., Dong M., Kiyota H., Gu Y.-C., Cong B. (2012). Constituents from *Salvia* Species and Their Biological Activities. Chem. Rev..

[5] Craft J., Satyal P., Setzer W. (2017). The Chemotaxonomy of Common Sage (*Salvia officinalis*) Based on the Volatile Constituents. Medicines.

[6] Leontaritou P., Lamari F.N., Papasotiropoulos V., Iatrou G. (2020). Morphological, Genetic and Essential Oil Variation of Greek Sage (*Salvia fruticosa* Mill.) Populations from Greece. Ind. Crops Prod..

[7] Leontaritou P., Lamari F.N., Papasotiropoulos V., Iatrou G. (2021). Exploration of Genetic, Morphological and Essential Oil Variation Reveals Tools for the Authentication and Breeding of *Salvia pomifera* Subsp. Calycina (Sm.) Hayek. Phytochemistry.

[8] Sharifi-Rad M., Ozcelik B., Altın G., Daşkaya-Dikmen C., Martorell M., Ramírez-Alarcón K., Alarcón-Zapata P., Morais-Braga M.F.B., Carneiro J.N.P., Alves Borges Leal A.L. (2018). *Salvia* Spp. Plants-from Farm to Food Applications and Phytopharmacotherapy. Trends Food Sci. Technol..

[9] Rivera D., Obon C., Cano F. (1994). The Botany, History and Traditional Uses of Three-Lobed Sage (*Salvia fruticosa* Miller) (Labiatae). Econ. Bot..

[10] Dimopoulos P., Raus T., Bergmeier E., Constantinidis T., Iatrou G., Kokkini S., Strid A., Tzanoudakis D. (2013). Vascular Plants of Greece: An Annotated Checklist.

[11] Sarrou E., Martens S., Chatzopoulou P. (2016). Metabolite Profiling and Antioxidative Activity of Sage (*Salvia fruticosa* Mill.) under the Influence of Genotype and Harvesting Period. Ind. Crops Prod..

[12] Cvetkovikj I., Stefkov G., Acevska J., Stanoeva J.P., Karapandzova M., Stefova M., Dimitrovska A., Kulevanova S. (2013). Polyphenolic Characterization and Chromatographic Methods for Fast Assessment of Culinary Salvia Species from South East Europe. J. Chromatogr. A.

[13] Atwi M., Weiss E.-K., Loupassaki S., Makris D.P., Ioannou E., Roussis V., Kefalas P. (2016). Major Antioxidant Polyphenolic Phytochemicals of Three *Salvia* Species Endemic to the Island of Crete. J. Herbs Spices Med. Plants.

[14] Koutsoulas A., Čarnecká M., Slanina J., Tóth J., Slaninová I. (2019). Characterization of Phenolic Compounds and Antiproliferative Effects of Salvia Pomifera and *Salvia fruticosa* Extracts. Molecules.

[15] Vergine M., Nicolì F., Negro C., Luvisi A., Nutricati E., Annunziata Accogli R., Sabella E., Miceli A. (2019). Phytochemical Profiles and Antioxidant Activity of *Salvia* Species from Southern Italy. Rec. Nat. Prod..

[16] Assessment Report on *Salvia fruticosa* Mill., Folium. https://www.ema.europa.eu/en/documents/herbal-report/final-assessment-report-salvia-fruticosa-mill-folium_en.pdf.

[17] Dawra M., Nehme N., Rayess Y.E., Beyrouthy M.E., Taillandier P., Bouajila J. (2022). Folk Medicinal Applications, Phytochemical Composition and Biological Activities of Some Lebanese Endemic Plants. South Afr. J. Bot..

[18] Bonesi M., Loizzo M.R., Acquaviva R., Malfa G.A., Aiello F., Tundis R. (2017). Anti-Inflammatory and Antioxidant Agents from *Salvia* Genus (Lamiaceae): An Assessment of the Current State of Knowledge. Anti-Inflamm. Anti-Allergy Agents Med. Chem..

[19] Stagos D., Portesis N., Spanou C., Mossialos D., Aligiannis N., Chaita E., Panagoulis C., Reri E., Skaltsounis L., Tsatsakis A.M. (2012). Correlation of Total Polyphenolic Content with Antioxidant and Antibacterial Activity of 24 Extracts from Greek Domestic Lamiaceae Species. Food Chem. Toxicol..

[20] Duletić-Laušević S.N., Alimpić Aradski A.Z., Kolarević S.M., Vuković-Gačić B.S., Oalđe M.M., Marin P.D. (2018). Biological Activities of Cretan Salvia Pomifera Extracts. Bot. Serbica.

[21] Couladis M., Tzakou O., Verykokidou E., Harvala C. (2003). Screening of Some Greek Aromatic Plants for Antioxidant Activity. Phytother. Res..

[22] Vistoli G., De Maddis D., Cipak A., Zarkovic N., Carini M., Aldini G. (2013). Advanced Glycoxidation and Lipoxidation End Products (AGEs and ALEs): An Overview of Their Mechanisms of Formation. Free Radic. Res..

[23] Ben Khedher M.R., Hafsa J., Haddad M., Hammami M. (2020). Inhibition of Protein Glycation by Combined Antioxidant and Antiglycation Constituents from a Phenolic Fraction of Sage (*Salvia officinalis* L.). Plant Foods Hum. Nutr..

[24] Jung H.A., Park J.J., Min B.S., Jung H.J., Islam M.N., Choi J.S. (2015). Inhibition of Advanced Glycation Endproducts Formation by Korean Thistle, *Cirsium maackii*. Asian Pac. J. Trop. Med..

[25] Veličković D.T., Milenović D.M., Ristić M.S., Veljković V.B. (2006). Kinetics of Ultrasonic Extraction of Extractive Substances from Garden (*Salvia officinalis* L.) and Glutinous (*Salvia glutinosa* L.) Sage. Ultrason. Sonochem..

[26] Pitarokili D., Tzakou O., Couladis M., Verykokidou E. (1999). Composition and Antifungal Activity of the Essential Oil of *Salvia Pomifera* Subsp. Calycina Growing Wild in Greece. J. Essent. Oil Res..

[27] Karousou R., Vokou D., Kokkini S. (1998). Distribution and Essential Oils of Salvia Pomifera Subsp. Pomifera (Labiatae) on the Island of Crete (S Greece). Biochem. Syst. Ecol..

[28] Zgheib R., Yassine C., Azzi-Achkhouty S., Beyrouthy M.E. (2019). Investigation of Essential Oil Chemical Polymorphism of *Salvia Fruticosa* Naturally Growing in Lebanon. J. Essent. Oil Bear. Plants.

[29] Skoula M., Hilali I.E., Makris A.M. (1999). Evaluation of the Genetic Diversity of Salvia Fruticosa Mill. Clones Using RAPD Markers and Comparison with the Essential Oil Profiles. Biochem. Syst. Ecol..

[30] Zimmermann B.F., Walch S.G., Tinzoh L.N., Stühlinger W., Lachenmeier D.W. (2011). Rapid UHPLC Determination of Polyphenols in Aqueous Infusions of *Salvia officinalis* L. (Sage Tea). J. Chromatogr. B.

[31] Martins N., Barros L., Santos-Buelga C., Henriques M., Silva S., Ferreira I.C.F.R. (2015). Evaluation of Bioactive Properties and Phenolic Compounds in Different Extracts Prepared from *Salvia officinalis* L.. Food Chem..

[32] Gulsoy Toplan G., Kurkcuoglu M., Goger F., İşcan G., Ağalar H.G., Mat A., Baser K.H.C., Koyuncu M., Sarıyar G. (2017). Composition and Biological Activities of Salvia Veneris Hedge Growing in Cyprus. Ind. Crops Prod..

[33] Wang M., Li J., Rangarajan M., Shao Y., LaVoie E.J., Huang T.-C., Ho C.-T. (1998). Antioxidative Phenolic Compounds from Sage ( *Salvia Officinalis* ). J. Agric. Food Chem..

[34] Borrás Linares I., Arráez-Román D., Herrero M., Ibáñez E., Segura-Carretero A., Fernández-Gutiérrez A. (2011). Comparison of Different Extraction Procedures for the Comprehensive Characterization of Bioactive Phenolic Compounds in Rosmarinus Officinalis by Reversed-Phase High-Performance Liquid Chromatography with Diode Array Detection Coupled to Electrospray Time-of-Flight Mass Spectrometry. J. Chromatogr. A.

[35] Liu M., Li Y.-G., Zhang F., Yang L., Chou G.-X., Wang Z.-T., Hu Z.-B. (2007). Chromatographic Fingerprinting Analysis of Danshen Root (*Salvia miltiorrhiza* Radix et Rhizoma) and Its Preparations Using High Performance Liquid Chromatography with Diode Array Detection and Electrospray Mass Spectrometry (HPLC-DAD-ESI/MS). J. Sep. Sci..

[36] Tada M., Hara T., Hara C., Chiba K. (1997). A Quinone Methide from *Salvia officinalis*. Phytochemistry.

[37] Trikka F.A., Nikolaidis A., Ignea C., Tsaballa A., Tziveleka L.-A., Ioannou E., Roussis V., Stea E.A., Božić D., Argiriou A. (2015). Combined Metabolome and Transcriptome Profiling Provides New Insights into Diterpene Biosynthesis in *S. pomifera* Glandular Trichomes. BMC Genom..

[38] Zhang Y., Smuts J.P., Dodbiba E., Rangarajan R., Lang J.C., Armstrong D.W. (2012). Degradation Study of Carnosic Acid, Carnosol, Rosmarinic Acid, and Rosemary Extract ( *Rosmarinus officinalis* L.) Assessed Using HPLC. J. Agric. Food Chem..

[39] Maietta M., Colombo R., Corana F., Papetti A. (2018). Cretan Tea ( *Origanum dictamnus* L.) as a Functional Beverage: An Investigation on Antiglycative and Carbonyl Trapping Activities. Food Funct..

[40] Monnier V.M. (1990). Nonenzymatic Glycosylation, the Maillard Reaction and the Aging Process. J. Gerontol..

[41] Matsuura N., Aradate T., Sasaki C., Kojima H., Ohara M., Hasegawa J., Ubukata M. (2002). Screening System for the Maillard Reaction Inhibitor from Natural Product Extracts. J. Health Sci..

[42] Derbré S., Gatto J., Pelleray A., Coulon L., Séraphin D., Richomme P. (2010). Automating a 96-Well Microtiter Plate Assay for Identification of AGEs Inhibitors or Inducers: Application to the Screening of a Small Natural Compounds Library. Anal. Bioanal. Chem..

[43] Sasaki K., Chiba S., Yoshizaki F. (2014). Effect of Natural Flavonoids, Stilbenes and Caffeic Acid Oligomers on Protein Glycation. Biomed. Rep..

[44] Ou J., Huang J., Wang M., Ou S. (2017). Effect of Rosmarinic Acid and Carnosic Acid on AGEs Formation in Vitro. Food Chem..

