# Supplementary material for: GC-MS and LC-DAD-MS Phytochemical Profiling for Characterization of Three Native Salvia Taxa from Eastern Mediterranean with Antiglycation Properties
Source: Molecules. 2022 Dec 22;28(1):93. doi: 10.3390/molecules28010093 (PMC9821822; doi:10.3390/molecules28010093)
Supplement: Supplementary file 1 [file molecules-28-00093-s001.zip › molecules-2055741-supplementary.pdf]

molecules

MDPI

Supplementary Information

GC-MS and LC-DAD-MS phytochemical profiling for characterization of three native *Salvia* taxa from Eastern Mediterranean with antiglycation properties

Maria D. Gkioni [1], Konstantina Zeliou [1], Virginia D. Dimaki [1], Panayiotis Trigas [2] and Fotini N. Lamari [1,*]

[1] Laboratory of Pharmacognosy & Chemistry of Natural Products, Department of Pharmacy, School of Health Sciences, University of Patras, 26504 Patras, Greece

[2] Laboratory of Systematic Botany, Department of Crop Science, School of Plant Sciences, Agricultural University of Athens, Iera Odos 75, 118 55, Athens, Greece

*Correspondence: flam@upatras.gr; Tel.: +30-2610-962335

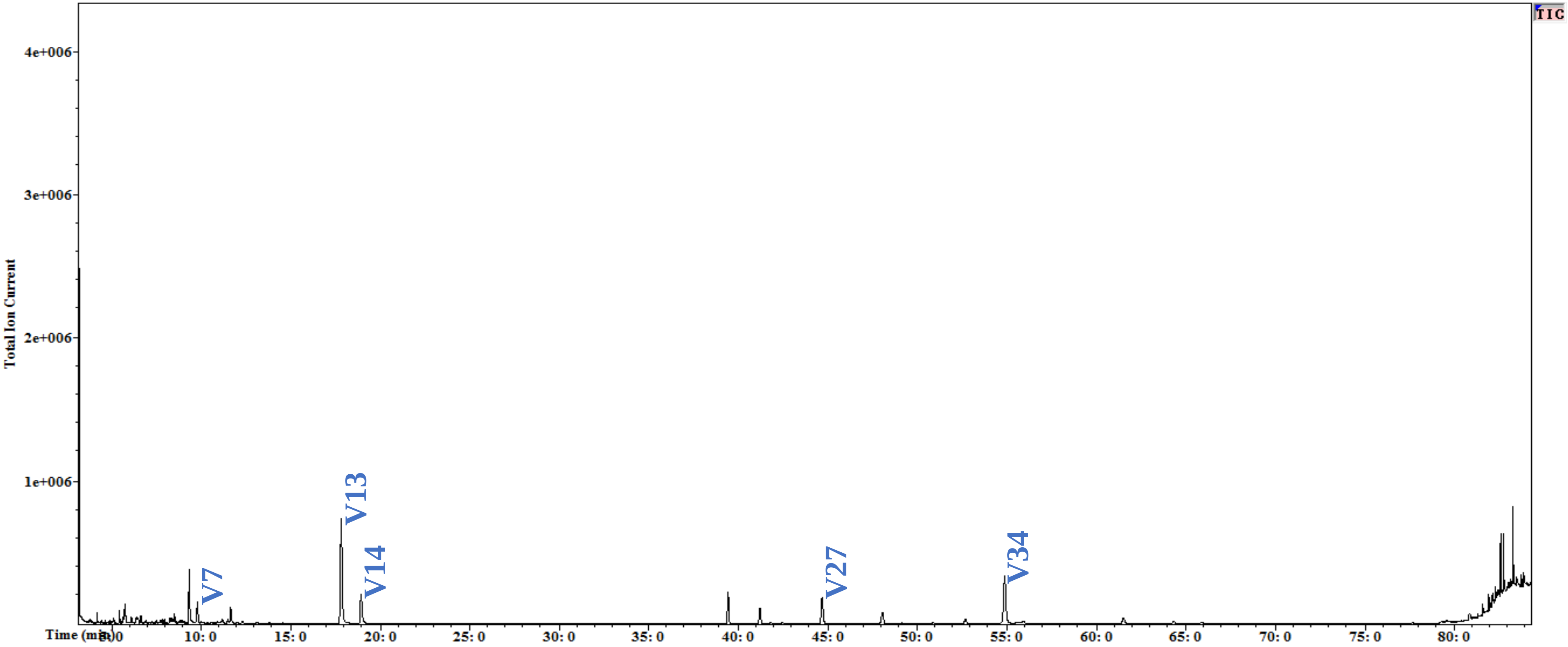

Figure S1. Representative total ion GC chromatogram of the petroleum ether extract of the leaves of the population **SPC-A**.

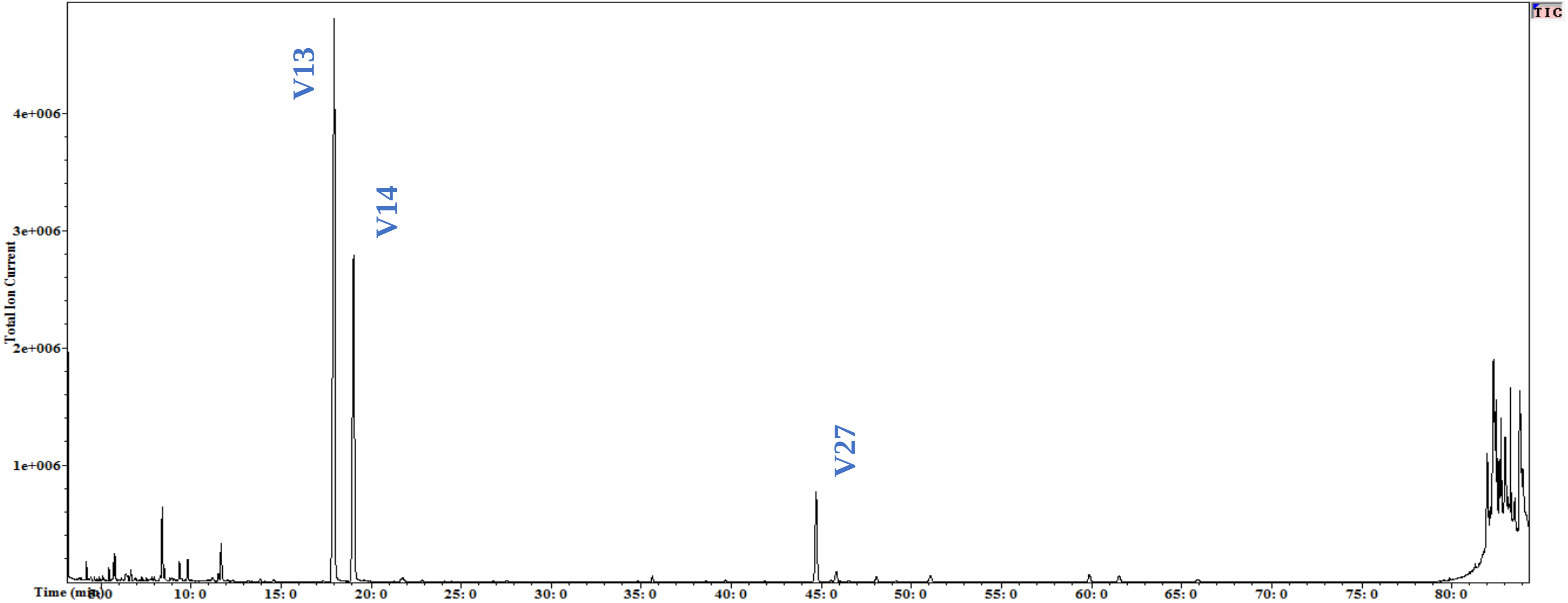

Figure S2. Representative total ion GC chromatogram of the petroleum ether extract of the leaves of the population **SPP-E**.

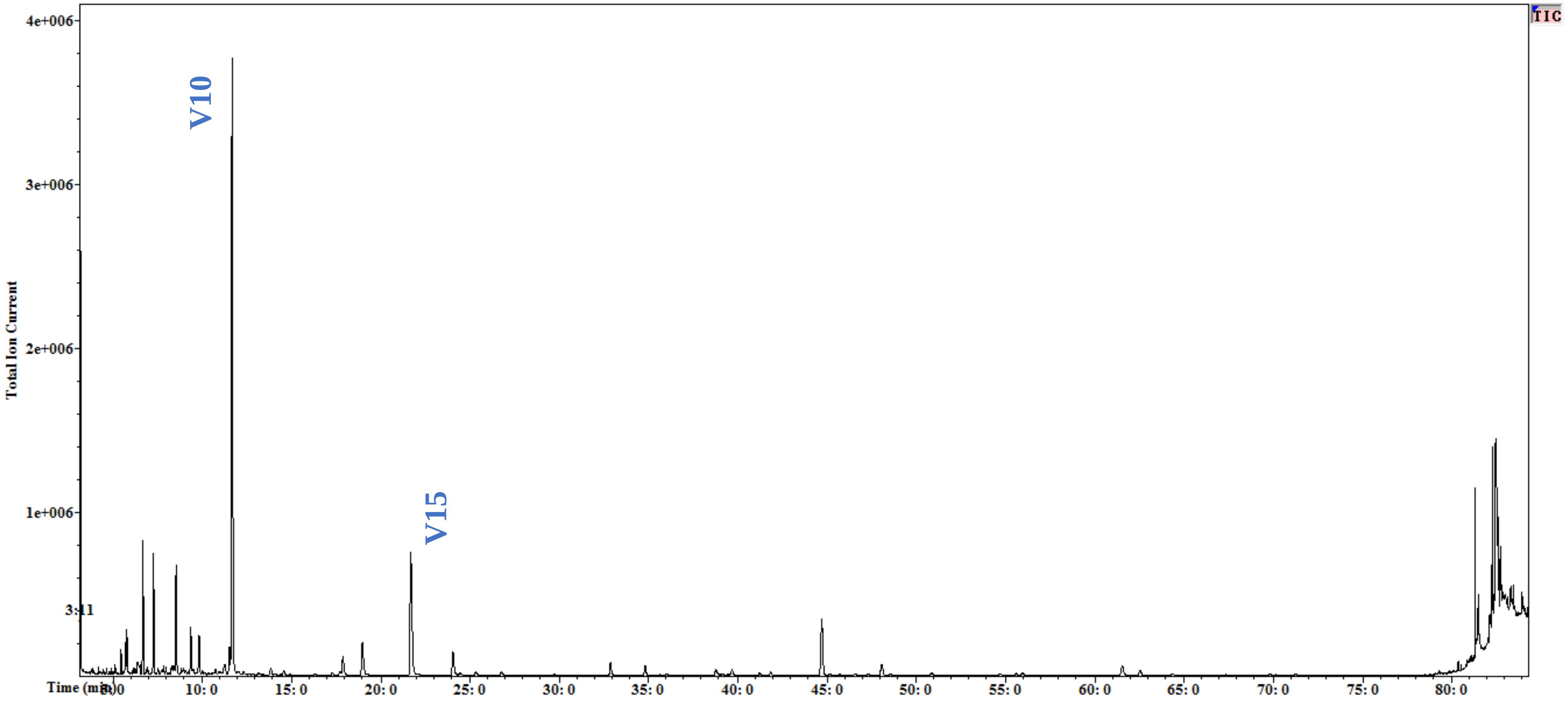

Figure S3. Representative total ion GC chromatogram of the petroleum ether extract of the leaves of the population **SF-S.**

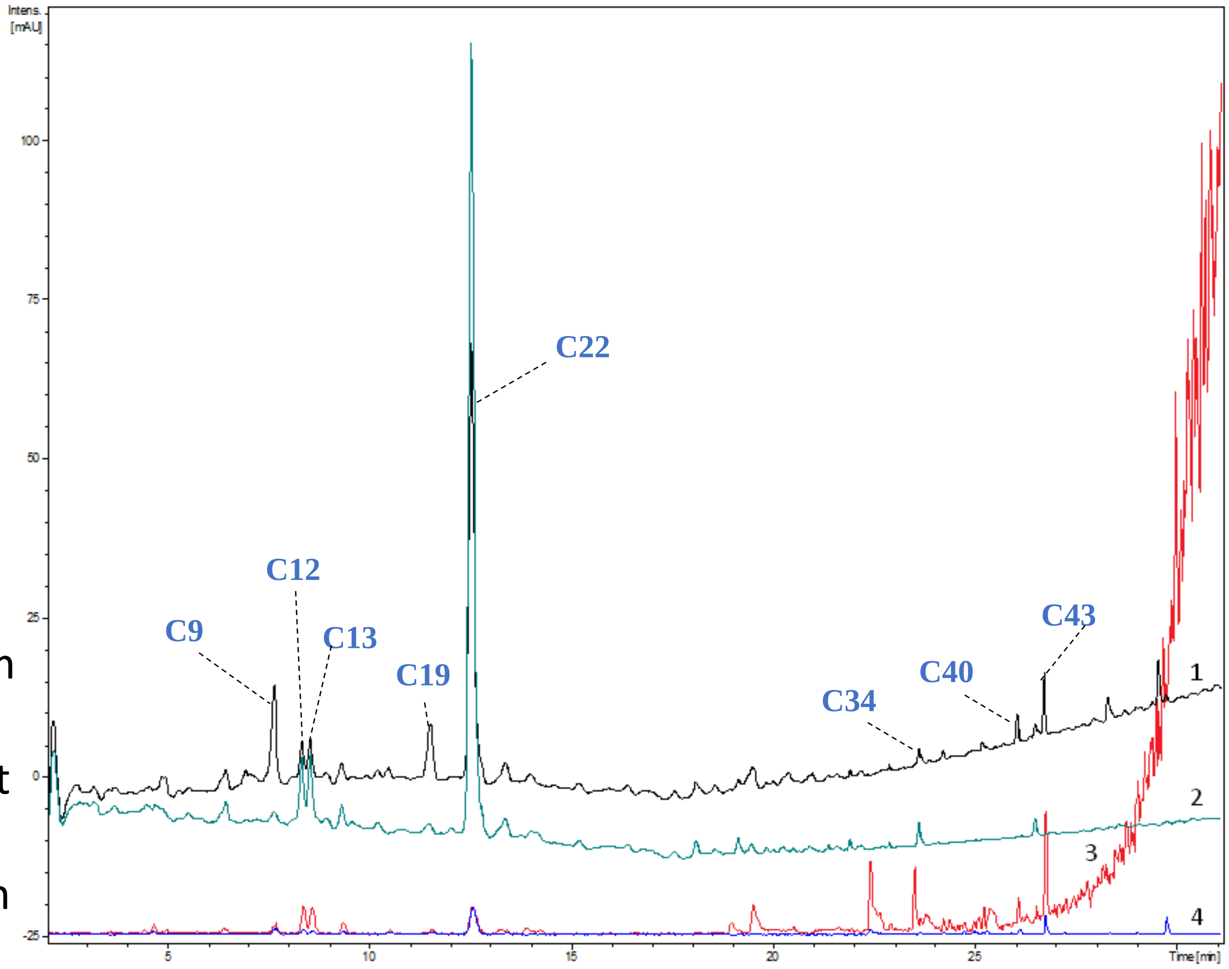

Figure S4. Representative chromatogram from the UHPLC-DAD-MS analysis of the hydroalcoholic extract of the leaves of the population **SPC-A** at 280 nm (1, black), at 330 nm (2, green), the total ion current after positive ionization (3, red) and negative ionization (4, blue).

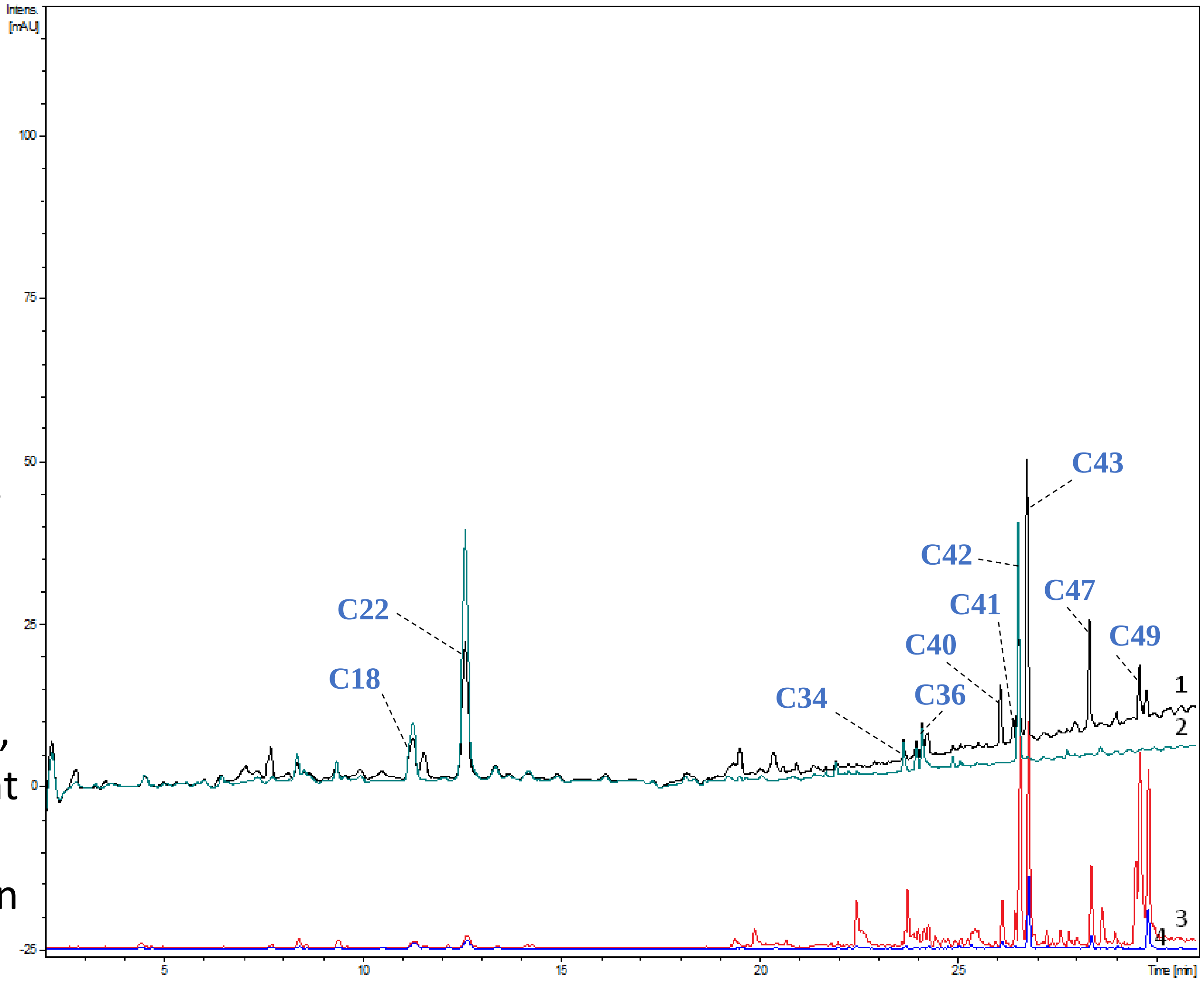

Figure S5. Representative chromatogram from the UHPLC-DAD-MS analysis of the hydroalcoholic extract of the leaves of the population **SPP-FS** at 280 nm (1, black), at 330 nm (2, green), the total ion current after positive ionization (3, red) and negative ionization (4, blue).

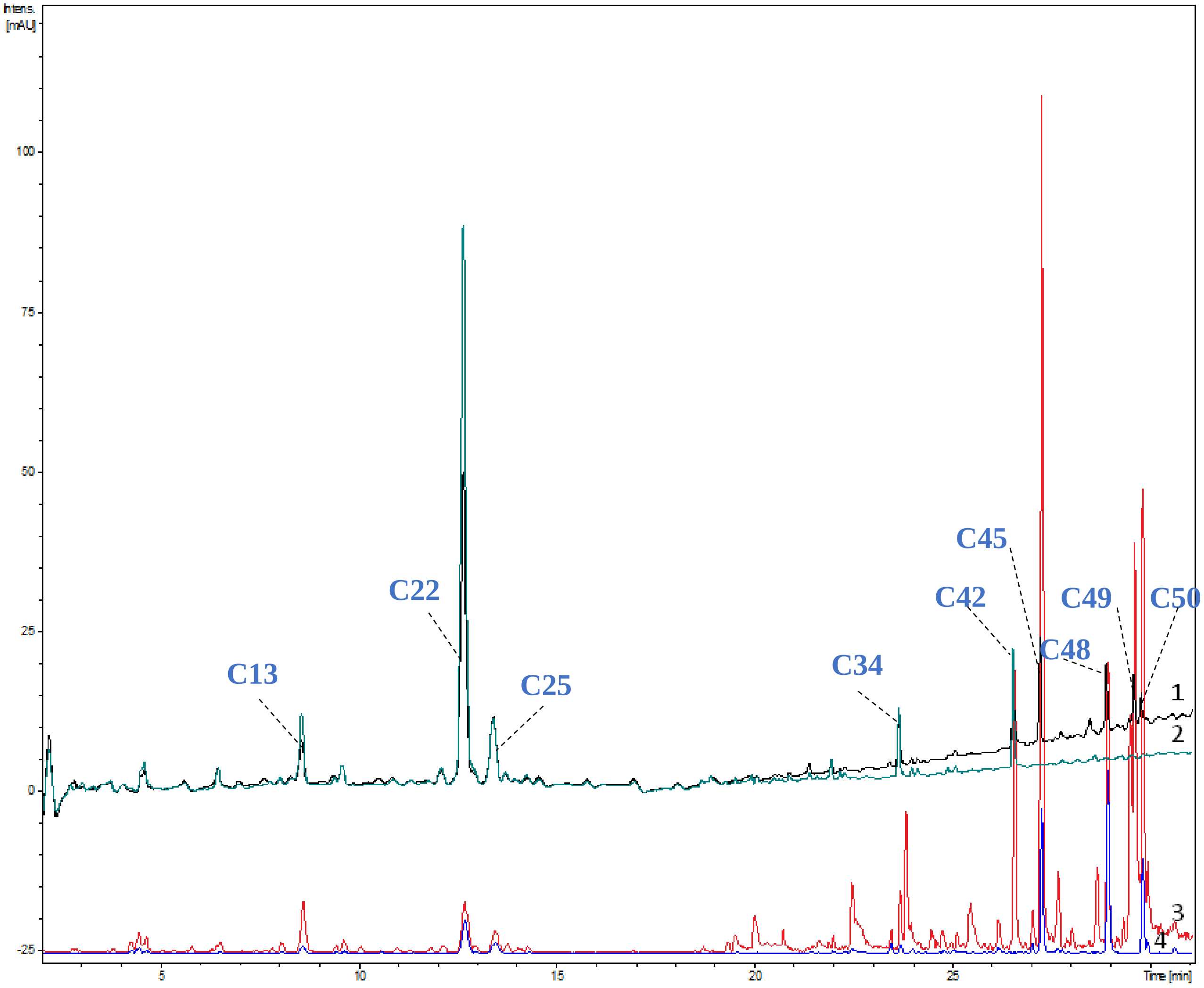

Figure S6. Representative chromatogram from the UHPLC-DAD-MS analysis of the hydroalcoholic extract of the leaves of the population **SF-S** at 280 nm (1, black), at 330 nm (2, green), the total ion current after positive ionization (3, red) and negative ionization (4, blue).

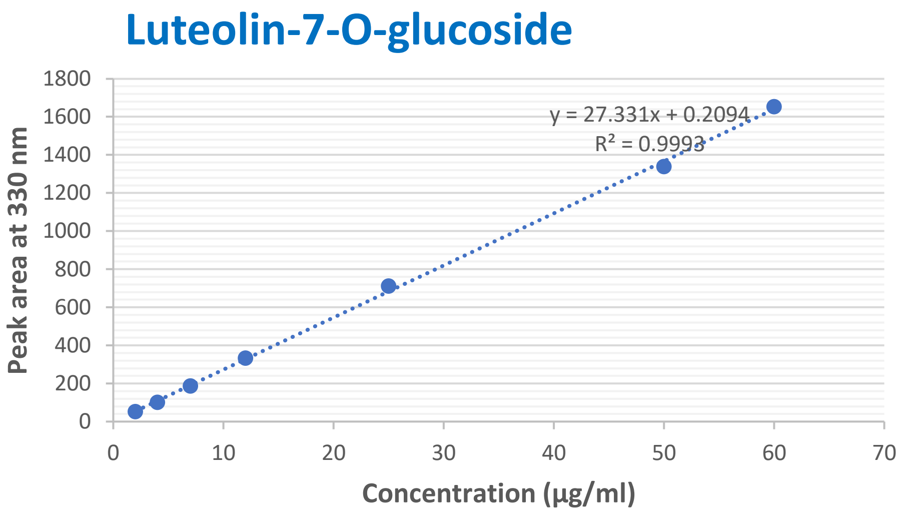

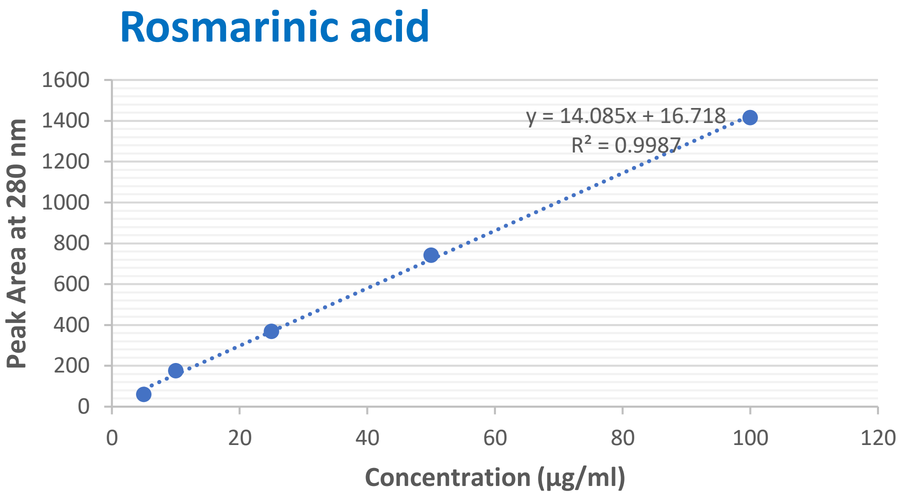

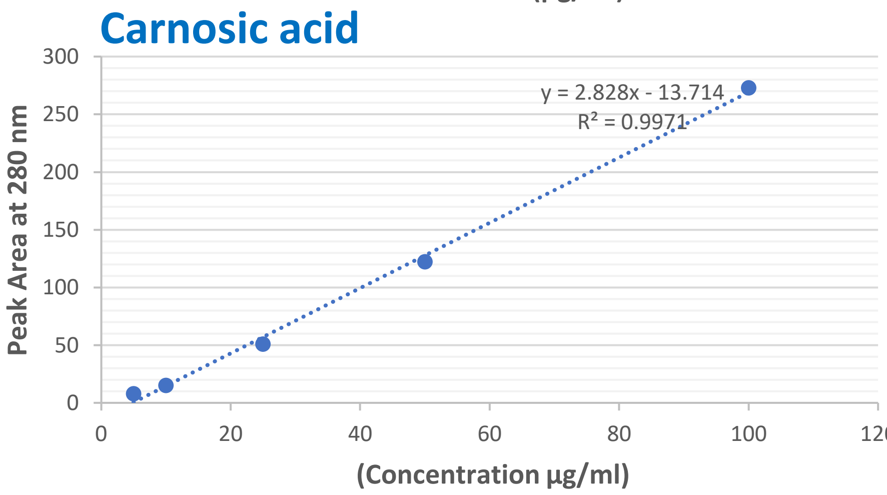

Figure S7. Linearity calibration curves of luteolin-7-O-glucoside, rosmarinic acid and carnosic acid that were used as external standards in the UHPLC-DAD-MS analysis of the hydroalcoholic extracts of the leaves of the various *Salvia* taxa. Each one was used for a different category of compounds.
